# Beyond the Numbers: Interpreting WHO's *Global Tuberculosis Report 2016* to Inform TB Policy and Practice in the East African Community

**DOI:** 10.24248/EAHRJ-D-16-00364

**Published:** 2017-03-01

**Authors:** Wilber Sabiiti, on behalf of the TWENDE consortium

**Affiliations:** a University of St. Andrews, St. Andrews, UK; b TWENDE consortium: University of St. Andrews (Dr Wilber Sabiiti, Prof. Stephen H Gillespie, and Dr Ewan Chirnside); Kilimanjaro Clinical Research Institute, Moshi, Tanzania (Prof. Blandina Mmbaga); Mbeya Medical Research Centre—National Institute of Medical Research, Mbeya, Tanzania (Dr Nyanda Elias Ntinginya); Kenya Medical Research Institute, Nairobi, Kenya (Dr Evans Amukoye); Makerere University, Kampala, Uganda (Dr Alphonse Okwera and Prof. Moses Joloba); CPAR Uganda Ltd, Lira, Uganda (Ms Norah Owaraga); and East African Health Research Commission, Arusha, Tanzania (Prof. Gibson Sammy Kibiki)

## Abstract

By 2000, 5 East African Community (EAC) member states—Uganda, Kenya, Tanzania, Rwanda, and Burundi—had adopted the World Health Organization's (WHO's) policy of directly observed treatment short-course (DOTS) for tuberculosis (TB). This policy is meant to speed up the control of TB through effective diagnosis and treatment. However, the rate of reduction of TB burden has been slow, and as of 2016, 3 EAC member states—Uganda, Kenya, and Tanzania—are still categorised as high TB burden countries. We analysed WHO's *Global Tuberculosis Report 2016* and drew key lessons to inform policy and practice for effective control of TB. From the report, we acknowledge the existence of national TB control policies operationalised through national TB control programmes in all EAC member states. However, we found persistent underfinancing of the TB control programmes; low national coverage of TB diagnostic and treatment services, meaning that many TB cases are most likely going undetected; and deaths due to lack of treatment. We also found poor reporting practices; for example, there was no data on the number of cases detected with rapid diagnostics in Uganda and Tanzania, which was unexpected since there are more than 170 Xpert MTB/RIF machines for rapid diagnosis of TB in the 2 countries. We recommend comprehensive implementation of existing TB policy, including adequate financing, universal access to diagnosis and treatment, and socioeconomic empowerment of affected communities, all of which are critical for ending TB in East Africa and the world at large.

## INTRODUCTION

The World Health Organization (WHO) released its most recent global tuberculosis (TB) report in October 2016.^1^ This annual report outlines achievements and challenges in the global control of TB in the preceding year and sets out goals and strategies for TB control programmes.^2^ The 2016 report reiterates the vision for global elimination of TB by 2035, but also sets out a phased progress evaluation programme focusing on reducing incidence of TB by 20% in 2020, then 50% in 2025, 80% in 2030, and finally 90% (with 95% reduction in TB deaths) in 2035.^3^

The global rate of reduction in TB burden was slow and did not change between 2014 and 2015, which has raised concerns about achieving the 2035 vision. The 2016 report listed 30 high TB burden countries that will be closely monitored for progress and achievement of the end TB targets. Three of the 6 member states of the East African Community (EAC)—Uganda, Kenya, and Tanzania—are on this high TB burden list. (As of 2016, the six member states of the EAC are Burundi, Kenya, Rwanda, South Sudan, Tanzania, and Uganda.) The period from 2016 to 2020 will mark the first phase during which these countries will be monitored for progress towards achieving a 20% reduction of non-HIV-associated, HIV-associated, and drug-resistant TB. We analysed WHO's *Global Tuberculosis Report 2016* and recommend policy action points that could be taken by the EAC to accelerate the end of TB in the region.

## TB BURDEN, INCIDENCE, AND MORTALITY IN THE EAC

The *Global Tuberculosis Report 2016* shows that the EAC has a combined TB burden of 388,600 cases (see Table 1). This represents 0.23% of the total EAC population of 173 million. There were 91,340 deaths in 2015, which is 24% of the TB cases across the EAC. This rate of mortality is unacceptably high, and it has never been more urgent to find more effective interventions to prevent such deaths. In addition, 32% (124,800) of the TB cases were among people living with HIV (PLHIV). On average, TB prevalence among PLHIV was 25% across the EAC, with a prevalence of more than 30% in Uganda, Kenya, and Tanzania. This means that HIV infection remains the highest risk factor for developing TB disease in this region, and thus efforts to defeat TB must include defeating HIV too.

**TABLE 1.** Tuberculosis Burden in EAC Member States, 2015

Country	Population (Millions)	TB Cases (includes PLHIV)	Males as % of Total TB Cases	Annual TB Mortality (includes PLHIV)
Uganda	39	79,000	66%	11,900
Kenya	46	107,000	62%	16,200
Tanzania	53	164,000	61%	55,000
Rwanda	12	6,600	65%	720
Burundi	11	14,000	63%	3,360
South Sudan	12	18,000	72%	4,160
*Total*	*173*	*388,600*	—	*91,340*

Abbreviations: EAC, East African Community; PLHIV, people living with HIV; TB, tuberculosis. Note: Data from the World Health Organization's *Global Tuberculosis Report 2016*.

Important to note also is that more than 60% of the TB patients in each of these countries were men (Table 1). This observation concurs with reports from clinical trials, in which the majority of TB cases are men.^4,5^ On a global scale, however, more women than men die of TB. Further research should investigate the biological factors that may underlie different responses to TB infection among men and women, as well as behavioural and cultural practices that increase the risk of contracting TB or limit access to health care.^5–7^ The gender-based disparities associated with TB are comprehensively discussed in a 2015 United Nations Development Programme discussion paper, which also highlights actions to be undertaken.^7^

Apart from South Sudan, which came into existence as a nation in 2011, the other 5 EAC member states have seen a decrease in TB incidence and rate of mortality in the last 15 years. In 2000, the average TB incidence across the EAC was 290 per 100,000, and this number fell to 184 per 100,000 population in 2015. This represents a fall of 7.1 cases per 100,000 population per year over 15 years (Table 2). The slow decrease in incidence perhaps explains why Uganda, Kenya, and Tanzania are still on WHO's list of countries with high TB burden. Likewise the average rate of mortality due to non-HIV-associated TB fell from 41 to 24 per 100,000 population in 2015 across the original 5 EAC states. Although the general average shows a decrease in mortality rate, however, Kenya had a 2-point rise, from 18 to 20 deaths per 100,000 population in mortality due to non-HIV-associated TB (Table 2). The WHO report excludes HIV-associated TB mortality, which implies that the mortality rate may have been even higher than reported.

**TABLE 2.** Change in TB Incidence and Mortality Rate in EAC Member States, 2000–2015

	TB Incidence			TB-related Mortality		
Country	2000^a^	2015	Annual Decrease in Incidence	2000^a^	2015	Annual Decrease in Mortality
Uganda	300	202	6.5	50	14	2.4
Kenya	300	233	4.5	18	20^b^	−0.1
Tanzania	450	306	9.6	70	56	0.9
Rwanda	100	56	2.9	5	4	0.1
Burundi	300	122	11.9	60	24	2.4
South Sudan	150^a^	146	1.0	30^a^	28	0.5
Average	*290*	*184*	*6.1*	*41*	*24*	*1.0*

Notes: Numbers are per 100,000 population, excluding cases among people living with HIV. Decrease in incidence and decrease in mortality is the change divided by 15 years (4 years for the case of South Sudan). Data from the World Health Organization's *Global Tuberculosis Report 2016*.

a South Sudan's estimated numbers are from 2011, not 2000. 2001 is the year South Sudan entered the EAC.

b Kenya was the only EAC state to show an increase in mortality from 2000 to 2015.

### Drug-Resistant TB

The burden of drug-resistant TB is generally low within the EAC. Drug-resistant TB can be categorised as monoresistant—resistance to one anti-TB drug such as rifampicin; multidrug resistant (MDR)—resistance to both rifampicin and isoniaizid; polydrug resistant—resistance to more than one first-line anti-TB drug other than both isoniaizid and rifampicin; and last but not least, extensive drug resistant (XDR)—resistance to any fluoroquinolone and at least one of the 3 second-line injectable anti-TB drugs.^8^ The estimated burden of rifampicin-resistant (RR) TB is 2% (7,920/388,600) of the total TB burden in the EAC.

Only 48% of the estimated 7,920 MDR/RR-TB cases in the EAC were reported in 2015, and on average only 30% were confirmed by a laboratory test (Table 3). The low rates of MDR/RR-TB case notification and laboratory confirmation are most likely due to inadequate laboratory capacity for drug sensitivity testing in the EAC states. For example, there are only 3 laboratories with drug sensitivity testing capacity in Tanzania, 4 in Kenya (3 of which are in the Nairobi area), and 3 in Uganda (all of which are in Kampala). This could be a recent improvement, however, as the 2015 version of the WHO global TB report shows only 1 drug sensitivity testing laboratory in Kenya and Uganda.^3^ Also important to note is that in all 6 EAC states, the ratio of MDR/RR-TB to total TB cases, although low among new cases, was high among patients with previous exposure to anti-TB drugs (Table 3). It should become a priority for every TB control programme to ensure that patients (1) are prescribed a suitable regimen and dose, and (2) complete their treatment, to curtail the emergence of more drug-resistant strains of TB. Better treatment response monitoring tools are crucial to check effectiveness of prescribed therapy.^9,10^

**TABLE 3.** Burden of Drug-Resistant TB in EAC Member States, 2015

Country	Estimated Burden of MDR/RR-TB	New MDR/RR-TB Cases Notified	New Cases (% of Total)	Previously Treated Cases (% of Total)	Laboratory-Confirmed Cases (% of Total)
Uganda	1,900	1,000	2%	12%	25%
Kenya	2,000	1,400	1%	9%	26%
Tanzania	2,600	730	1%	30%	24%
Rwanda	160	120	2%	11%	78%^a^
Burundi	500	190	3%	14%	23%
South Sudan	760	370	7%	8%	5%
*Total*	*7,920*	*3,810*	*3*%^b^	*14*%^b^	*30*%^b^

Abbreviations: EAC, East African Community; MDR-TB, multidrug-resistant TB; RR-TB, rifampicin-resistant TB; TB, tuberculosis. Notes: Data from the World Health Organization's *Global Tuberculosis Report 2016*. Previously treated patients are at higher risk of developing drug-resistant tuberculosis.

a Rwanda was the only EAC state with a rate of laboratory-confirmed drug-resistant TB greater than 30%.

b Values are average percentages.

### TB Diagnosis

Strong laboratory services are necessary to ensure accuracy of diagnosis and prescription. WHO's *Global Tuberculosis Report 2016* shows low coverage of rapid tests, although more than 50% of the notified TB cases in all EAC states were bacteriologically confirmed (Table 4). A bacteriologically confirmed case is one that has tested positive for TB by either smear microscopy or culture or by rapid molecular tests such as Xpert MTB/RIF and line probe assay.^8^ Rapid tests give results in a matter of hours, simultaneously detecting the presence of TB and drug resistance.^11^ Low coverage of rapid tests means that TB testing still largely depends on smear microscopy, a method that has been shown to be poor at detecting TB in low-burden patients, particularly children and PLHIV.^12^ Coverage of bacterial culture for TB diagnosis and drug sensitivity testing is still low, on average 1 per 5 million population, and thus cannot account for a large proportion of the bacteriologically confirmed cases.^3^

No data on rapid tests was given for Uganda or Tanzania, despite that there are 111 and 74 Xpert MTB/RIF machines, respectively, in these 2 countries.^3^ This raises concern over the quality of data collection and reporting, which needs to be addressed. Data quality is crucial to control of TB.^13^ Across EAC states, an average of 89% of TB cases had known HIV status, which indicates that the region has a higher testing capacity for HIV than for TB (Table 4).

**TABLE 4.** Proportion of Total Notified TB Cases Tested for TB and HIV, 2015

Country	Total Case Notification 2015	% Confirmed With Rapid Tests	% Confirmed Bacteriologically	% With Known HIV Status
Uganda^a^	43,736	—	71%	91%
Kenya	81,518	10%	59%	82%
Tanzania^a^	62,180	—	53%	92%
Rwanda	5,637	39%	86%	96%
Burundi	6,969	8%	88%	95%
South Sudan	10,250	2%	56%	79%
*Average*	*35,048*	*15%*	*69%*	*89%*

Note: Data from the World Health Organization's *Global Tuberculosis Report 2016*.

a Data for the percentage of cases tested by rapid diagnostic tests in Uganda and Tanzania was missing.

### TB Treatment

The purpose of diagnosis is not accomplished if there is no treatment, and treatment success begins with accessibility. On average, 60% of the EAC has TB treatment coverage. This means that 40% of the population of the EAC has hardly any access to TB treatment. Rwanda posted the highest rate of treatment coverage at 84%, whereas the lowest rate was in Tanzania, at 37% (Table 5). Despite having the least treatment coverage, Tanzania and Burundi achieved 90% treatment success among new TB cases. However, the low treatment coverage in these countries means that many patients may have gone untreated and died. Overall treatment success was lower among previously treated, HIV-associated, and drug-resistant TB cases (Table 5). While recognising the great treatment success achieved among those patients who had access to treatment, the policy ambition should be to expand TB treatment coverage to 100% of the population.

**TABLE 5.** TB Treatment Coverage by Country and TB Treatment Success by Patient Cohort, 2015

		Treatment Success Rate (%, *n*)				
Country	TB Treatment Coverage	New and Relapsed Cases	Previously Treated Cases	Cases Among PLHIV	MDR/RR-TB Cases	XDR-TB Cases
Uganda	57%	75% (43,628)	67% (2,438)	73% (16,670)	73% (214)	0% (0)
Kenya	76%	87% (89,294)	78% (222)	82% (30,107)	82% (266)	0% (1)
Tanzania	37%	90% (61,573)	81% (1,578)	87% (20,658)	68% (92)	0% (0)
Rwanda	84%	86% (5,846)	80% (94)	—	81% (94)	0% (0)
Burundi	51%	91% (7,309)	82% (83)	86% (901)	89% (38)	0% (0)
South Sudan	54%	71% (8,335)	69% (521)	71% (859)	—	—
*Average*	*59%*	*83%*	*76%*	*80%*	*76%*	*0%*

Abbreviations: MDR-TB, multidrug-resistant TB; RR-TB, rifampicin-resistant TB; PLHIV, people living with HIV; TB,tuberculosis; XDR-TB, extensively drug-resistant TB. Note: Data from the World Health Organization's *Global Tuberculosis Report 2016*. In all countries, treatment success rate was lower among previously treated cases than among new cases. Treatment failed for the single XDR-TB case in Kenya.

## FINANCING OF TB CONTROL PROGRAMMES

All the EAC member states except Burundi had a budget for TB control programme activities in the 2016 financial year. Domestic funding constituted less than 25% in all countries. Unfunded budgets ranged from 0% in Kenya and Rwanda to 55% in Tanzania; although the budget was fully funded in Kenya and Rwanda, most of the funding came from external donors. Funding gaps are common, and EAC states have had yearly budget shortfalls almost every year since 2012, with the unfunded budget sometimes going up to 100% of the total (Table 6).

**TABLE 6.** Financing of TB Control Programme Activities by EAC Member State, 2015

Country	Budget 2016 (Million US$)	% Domestically Funded	% Externally Funded	% Unfunded 2016	% Unfunded 2012–2015
Uganda	38	4%	78%	19%	20%–80%
Kenya	59	20%	80%	0%	30%–60%
Tanzania	40	5%	40%	55%	60%–100%
Rwanda	15	21%	60%	19%	0%–10%
Burundi	0	—	—	—	20%–100%
South Sudan	12	10%	59%	31%	10%–80%

Note: Data from the World Health Organization's *Global Tuberculosis Report 2016*.

Going forward, the EAC must strive to increase domestic funding of national TB control programmes to assure citizens of these essential health care services. Bearing in mind the competing demands on the meagre resource envelopes of the EAC states, one realises that domestic funding in the short term can be found in savings made by fostering programme cooperation, such as between HIV and TB control programmes. This could include building well-coordinated coalitions of stakeholders for coordinated efficient action that results in larger impact. Coordination of local and international players will avoid duplication of interventions and save resources that can be invested elsewhere. Savings can be invested in ensuring a consistent supply of medicines and diagnostics, and in hiring and retaining skilled human resources. As in Rwanda, implementing affordable health insurance premiums could increase access to health care in other member states.^14^ Indeed, Rwanda's achievement of 84% TB treatment coverage was the highest in the region. All EAC member states should work towards achieving 15% expenditure on health, as recommended by the African Union. The long-term target should be providing quality health care at the nearest location to the patient and achieving universal health care coverage.

## RECOMMENDED ACTIONS TO END TB IN THE EAC

In summary, the following action points, drawn from WHO's *Global Tuberculosis Report 2016*, can enable the EAC to realise the 2035 vision for ending TB:

- Coordinate HIV and TB diagnosis and treatment, which is critical to defeating both diseases. The steps for achieving this coordination are clearly outlined by AVERT's HIV and TB coinfection programmes.^15^
- Invest in building surveillance and data collection capacity to ensure accurate disease burden estimates, which are useful for resource allocation planning.
- Strengthen and expand TB diagnostic and treatment services to cover all citizens. This includes providing the required infrastructure and human resources to deliver the services^16^ and investing in postregistration anti-TB-drug clinical trials and affordable diagnostic approaches.
- Implement deliberate socioeconomic empowerment programmes, including health insurance, to increase health care affordability and accessibility.^17,18^
- Commit to increasing domestic funding for health care and reduce overdependency on external donor support.
- Ensure that governments act as team players in harnessing resources by ensuring the coordination and cooperation of local and international stakeholders in health care provision.
- Invest in research that can inform policy. Research into the social determinants of disease and research into new diagnostics and treatments should be equally supported.^19^

The *Global Tuberculosis Report 2016* importantly noted that as far as research is concerned, the diagnostic technology landscape looks quite promising, with new diagnostic platforms, new drugs, and new vaccines in the pipeline. To achieve maximum benefit, the EAC states should, first, not sit and wait to consume finished products but instead actively participate in research to bring these diagnostics, medicines, and vaccines to the clinic. Secondly, EAC states should put in place channels for rapid translation of research into policy and practice. This will happen as long as policy makers go after research evidence from national and international sources to support policies they make.

Thirdly, EAC states should committedly invest in implementing TB policies. As noted regarding TB budget financing, some member states have fallen short of their TB control budget or have not financed it at all. Allocation of resources is what brings a policy to life. Without financing, policies remain ‘on the shelf’ and are as good as absent. No one benefits from an unimplemented policy.
